# Physical activity and dietary intake among Chinese pregnant women: an observational study

**DOI:** 10.1186/s12884-019-2452-y

**Published:** 2019-08-14

**Authors:** Mi Xiang, Jing Zhang, Huigang Liang, Zhiruo Zhang, Masayuki Konishi, Huanhuan Hu, Mio Nishimaki, Hyeon-Ki Kim, Hiroki Tabata, Hisao Shimizu, Takashi Arao, Shizuo Sakamoto

**Affiliations:** 1 School of Public Health, Shanghai Jiao Tong University, Shanghai, China; 2 Department of Business Information & Technology, Fogelman College of Business & Economics, University of Memphis, Memphis, USA; 3 Faculty of Sport Sciences, Waseda University, Saitama, Japan; 4 National Center for Global Health and Medicine, Tokyo, Japan; 5 Graduate School of Sport Sciences, Waseda University, Saitama, Japan; 6 Research Fellow of the Japan Society for the Promotion of Science, Tokyo, Japan

**Keywords:** Physical activity, Nutrition, Pregnancy, Chinese women

## Abstract

**Background:**

Physical activity (PA) and dietary intake are important modifiable factors associated with health outcomes. However, Chinese pregnant women’s PA and dietary intake are only vaguely understood. The aim of this study was to reveal the characteristics of PA and dietary intake of Chinese women in different trimesters as well as the associations between PA and dietary intake.

**Methods:**

This is a cross-sectional observational study. PA, dietary intake, and demographics of 1077 Chinese pregnant women were measured. The Chi-square test, Kruskal-Wallis test, multiple logistic regression, and multiple linear regression were used for data analysis.

**Results:**

About 57.1% of the participants met the international guideline for PA. Household activity and occupational activity contributed the most to the total PA, while sports/exercise contributed little. The mean energy intake of the participants was 2008 ± 748.0 kcal. Most participants had normal energy intake, but they obtained excessive energy from fat (mean = 41.7 ± 8.7%). PA was not found to be significantly associated with dietary intake. Further, the participants who were unemployed during pregnancy (OR = 0.72, 95% CI: 0.55–0.95; *p* < 0.05) or had no exercise habits before pregnancy (OR = 0.62, 95% CI: 0.47–0.80; *p* < 0.01) were less likely to meet the PA guideline. The participants in the third trimester (OR = 1.43, 95% CI: 1.03–1.99; *p* < 0.05) were more likely to meet the PA guideline compared to those in the first trimester. The older participants (> 30 years) showed higher dietary intake than the younger (< 25 years) participants (*p* < 0.01).

**Conclusions:**

The total PA of Chinese women during pregnancy mostly consists of household and occupational activities, but little sports/exercise. Starting exercise before pregnancy may help women achieve adequate PA during pregnancy. Moreover, these women consumed an excessive amount of fat and their diet intake varies by age.

## Background

Physical activity (PA) and dietary intake are among the most important lifestyle factors affecting both maternal and infant health, which can be modified to prevent excessive gestation weight gain and macrosomia and to reduce the risk of complications such as gestational diabetes mellitus and preeclampsia [1–3]. The American College of Obstetricians and Gynecologists (ACOG) guideline for PA and diet intake during pregnancy recommends pregnant women to follow a low-fat diet (< 30% of energy) [4] and engage in at least 30 min of moderate-intensity PA on most days of the week [5]. However, this recommendation was developed primarily based on the research involving Caucasian women in developed countries. Given that people of different races are affected differently by PA [6, 7], the ACOG guideline may not be suitable for Chinese women. However, to date no PA guideline has been specifically developed for Chinese women, and little is known regarding Chinese women’s PA and diet during pregnancy.

China has one of the largest populations of pregnant women in the world. Over 50% of Chinese women experience excessive GWG [8] and more than 10% of Chinese women had macrosomia [9]. To develop effective interventions for improving the health of Chinese women and infants, it is necessary to comprehensively examine Chinese women’s PA and dietary intake during pregnancy. Most previous studies are limited by observing only one trimester, using insufficient questionnaires of PA (e.g., no questions concerning household activity, which is a major activity for pregnant women), and lacking the measurement of PA intensity and type [10–12]. To address these limitations, it is necessary to characterize PA during all trimesters of Chinese women and consider different intensities and types of PA. In addition, although previous research already showed that PA and dietary intake are correlated in general adults [13, 14], few studies have examined their association in pregnant women. Identifying influencing factors of these two behaviors will also help to refine and improve the intervention efforts.

Therefore, the aim of this study was to 1) reveal the characteristics of PA and dietary behavior, 2) examine the association of PA with dietary intake, and 3) identify influencing factors of these modifiable factors in Chinese pregnant women.

## Methods

### Study design and participants

A cross-sectional study was conducted. From June 2014 to January 2016, we recruited participants from the Maternal and Child Health Hospital in Chengdu Wuhou, Sichuan province, China. Chengdu is a large second-tier city, which includes both rural and urban areas. Pregnant women were eligible to participate if they were aged over 20 years, with a singleton pregnancy, of the race of Han, and did not have any major chronic disease such as diabetes mellitus, hypertension, heart disease, chronic renal disease, or other diseases that would restrict PA before pregnancy. Almost all pregnant women who visited the hospital for their usual antenatal care at Nutrition Department during their first, second, and third trimesters of pregnancy (< 13 weeks, 13–28 weeks, and > 28 weeks, respectively) were recruited through direct contact by the investigators. All eligible subjects who consented to participate were informed about the study’s purpose and procedures and were instructed to complete the survey. During the study, five participants filled out the questionnaires simultaneously, so that if they had questions they could ask the investigator. The group size was limited to five to make sure each person had opportunities to get help from the investigators. This study was approved by the committee on research involving human subjects of Waseda University [2014–037] and all participants provided written informed consent.

### Measurements

#### Physical activity

The PPAQ, a validated instrument, was used to measure PA during pregnancy [15]. It examines duration, frequency, and intensity of the total PA during the current trimester of pregnancy. It includes 32 activities and participants select the best estimation of the amount of time spent for each activity (e.g., “none,” “less than half an hour per day,” “half to almost 1 hour per day,” “1 to almost 2 hours per day,” “2 to almost 3 hours per day,” or “3 or more hours per day”). The activities are classified by type: household/caregiving (13 activities), occupational (5 activities), and sports/exercise (8 activities). These activities are also rated by intensity: sedentary (< 1.5 METs), light (1.5–< 3.0 METs), moderate (3.0–6.0 METs), or vigorous (> 6.0 METs), where 1 MET is the metabolic equivalent of the energy expended at rest. The average energy expenditure per week for each activity was calculated by multiplying the weekly number of hours by each activity intensity, and activities of at least light intensity were summed to derive average MET-hours per week for total activity (METs-h/week) [15]. This study used the Chinese translation of the PPAQ. The details of reliability and validity of this instrument have been published elsewhere [16].

#### Dietary intake

The semi-quantitative FFQ was used to evaluate dietary behavior. The validity and reliability of the questionnaire have been examined by using a sample of Chinese pregnant women [17]. FFQ includes 10 food groups: cereals, meats, fish, eggs, beans, vegetables, fruits, nuts, milk and milk products and beverages. Participants were required to recall their usual frequency of consuming each food item in the past 3 months. Food intake frequency was measured using the following categories: per day, per week, per month, per three months, or never. Frequencies were calculated as the average of the upper and lower frequencies divided by the time interval such as per day, per week, or per month. Food models representing standard portion size for most of the food items were prepared to help participants estimate their usual consumption. Daily dietary intake was calculated as the multiplication of three components: the total grams of each food consumed each time, the frequency of consumption, and the amount of nutrient in each gram of that food. Dietary intakes were calculated based on a food composition database created for this study by combining Chinese Food Composition Tables [18]. The participants reported an excessive energy intake (> 5000 kcal/day) or an energy intake of < 500 kcal/day were excluded [14].

### Covariates and other measurements

Demographic information including maternal age (< 25, 25–30, or > 30 year), weeks of pregnancy, education (less than high school, high school graduate, or college degree or higher), monthly household income (< 4000, 4000–8000, or > 8000 RMB), employment status (yes or no), smokers before pregnancy (yes or no), alcohol drinkers habits before pregnancy (yes or no), smokers during pregnancy (yes or no), alcohol drinkers during pregnancy (yes or no), and exercise habits before pregnancy (> 3 times of regular exercises per week, not limited to any intensity and type; yes or no), was obtained during the survey. Pre-pregnancy weight and height were obtained from antenatal examination card records, which was self-reported by the participants when they registered their pregnancy in the first trimester.

### Statistical analysis

PA was categorized into two groups based on the international guideline for PA (≥150 min of moderate- or higher-intensity activity per week): meeting the guideline or not. Chi-square tests were used to compare proportions of meeting the guideline for PA among the three trimesters. The Kruskal-Wallis test was used to compare total PA and dietary intake during the three trimesters. To examine the association of PA with dietary intake, multiple linear regression was used. To examine the influencing factors of PA, multiple logistic regression was conducted. Since previous research and our data showed that most Chinese women derived excessive energy from fat (high fat intake) in diet, we also examined the influencing factors of fat intake for Chinese women using multiple linear regression. All of the covariates associated with PA and dietary intake (i.e., age, pre-pregnancy BMI, education, employment status, household income, and exercise habits before pregnancy) were included in the models. *P* < 0.05 was considered significant. The SPSS v.21 (IBM Corp., Armonk, NY) was used for all the analyses. We adhered to SROBE guidelines to report the results of this study.

## Results

A total of 1272 pregnant women were recruited. We excluded 48 participants who had incomplete demographic information, 99 participants who did not complete the food frequency questionnaire (FFQ), 10 participants with excessive energy intake (> 5000 kcal/day) [14], 14 participants who did not complete the pregnancy physical activity questionnaire (PPAQ), and 24 participants reporting more than 24 h of average daily PA. No participant reported an energy intake of < 500 kcal/day [14]. Finally, 1077 participants were included in the analysis. The included and excluded participants did not differ significantly with respect to age, pre-pregnancy body mass index (BMI), exercise habits, education, income, weeks of pregnancy, employment status, or household income.

Table 1 presents the demographic characteristics of all the participants. Their mean age was 29 ± 5 years. They cover all three trimesters: first trimester (*n* = 314, 29.2%), second trimester (*n* = 395, 36.7%), and third trimester (*n* = 368, 34.2%). Among the participants, 71.5% began pregnancy with a normal pre-pregnancy BMI, 42.5% received high school education or less, and 44.5% were employed. About 59.9% exercised habitually before pregnancy and slow walking was the most commonly reported form of exercise. All socio-demographic characteristics of the subjects are presented in Table 1.

**Table 1:**
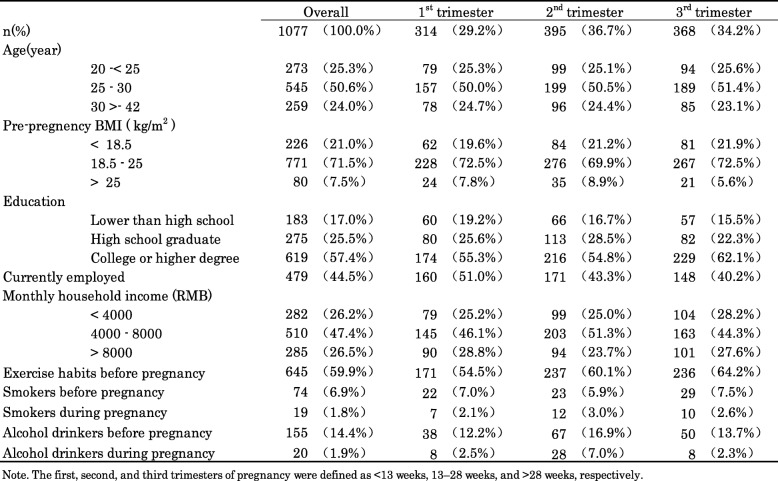
Socia-demographic characteristics of pregnant women n (%) (*n* = 1077)

The details of PA and energy intake during pregnancy in Chinese women are presented in Table 2. More than half (57.1%) of the participants met the international guideline for PA (≥150 mins of moderate-intensity PA per week). The median of their total PA was 56.3 METs-h/week. The participants in the third trimester had significantly higher median total energy expenditure (62.4 METs-h/week) than those in the first and second trimesters (52.8 and 54.9 METs-h/week, respectively; *p* = 0.008). Time spent on physical activities differed significantly between the trimester groups when PA was classified by type (*p* < 0.01), but not when classified by intensity except vigorous-intensity activity. In addition, the mean energy intake was 2008 ± 748.0 kcal, and varied significantly across the trimesters (ranging from 1838.6 to 2128.6 kcal, p < 0.01). The mean energy derived from fat was 41.7 ± 8.7%, over the guideline recommendation of below 30%. We also found that household activity and occupational activity contributed the most to the physical activity energy expenditure. The time spent on sports/exercise during the three trimesters ranged from 0.3 to 0.8 h/week, and the most reported exercise type was walking.

**Table 2:**
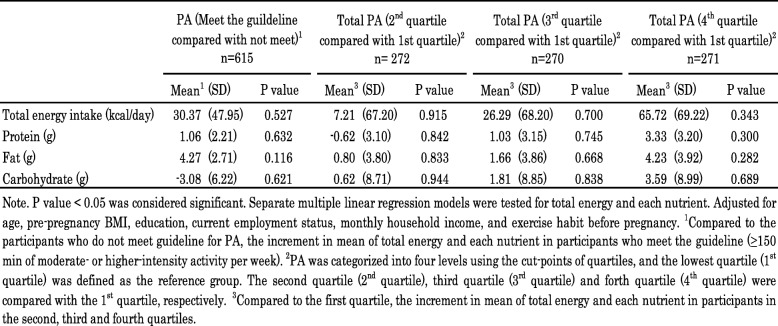
Physical activity and energy intake during pregnancy in chinese women (*n* = 1077)

We examined the relationship between PA and dietary intake during pregnancy by estimating daily mean intake of each food and nutrients within each PA level (Table 3). The participants with high level PA did not significantly intake a larger amount of total energy and nutrients.

**Table 3:**
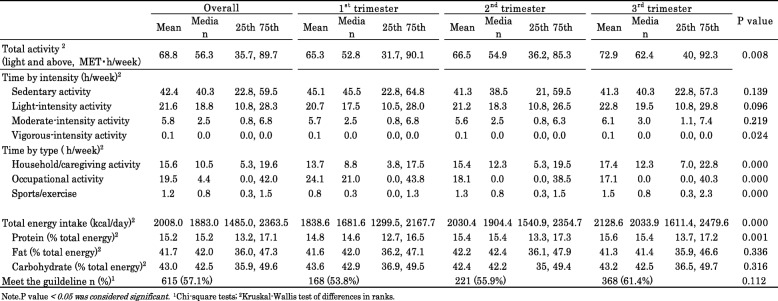
Increment in mean daily intake of total energy and each nutrients associated with specified level of PA

Table 4 presents the influence of socio-demographic factors on PA and dietary intake. The participants who were unemployed during pregnancy (OR = 0.72, 95% CI: 0.55–0.95; *p* < 0.05) were less likely to meet the PA guideline. The women without exercise habits before pregnancy were less likely to meet the PA guideline than those with exercise habits before pregnancy (OR = 0.62, 95% CI: 0.47–0.80; *p* < 0.01). The women in the third trimester (OR = 1.43, 95% CI: 1.03–1.99; *p* < 0.05) were more likely to meet the PA guideline than those in the first trimester. Dietary intake was associated with age and exercise habits before pregnancy. The participants older than 30 years showed a 2.5% increase in fat intake compared to those younger than 25 years (*p* < 0.01). Meanwhile, the women with exercise habits before pregnancy were more likely to intake excessive energy derived from fat (*p* < 0.01). Other variables such as education and pre-pregnancy BMI were not associated with PA or dietary intake.

**Table 4:**
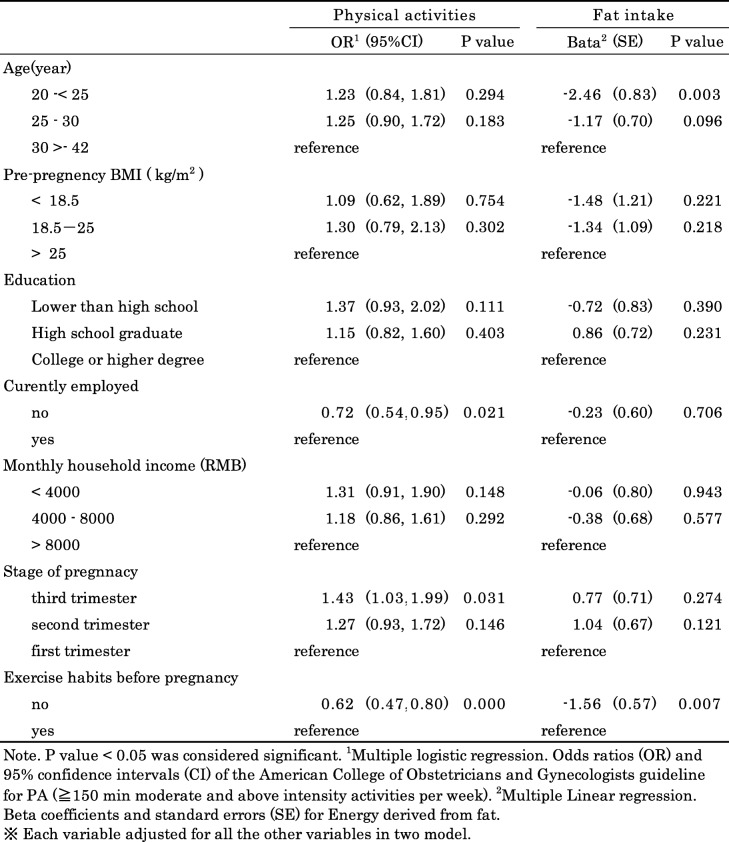
Sociodemographic and health behaviour predictor associated with physical activities and energy derived from fat during pregnancy (*n*-1077)

## Discussion

This is the first study to reveal lifestyle characteristics of pregnant Chinese women, considering multiple modifiable factors including PA intensities, PA types, and nutrients. In this study, we found Chinese pregnant women had relatively high total PA and more than half of them met the international guideline of PA. Their mean energy intake was within the normal range, but most of them derived excessive energy from fat. PA was not directly associated with the dietary intake behavior among the Chinese pregnant women. In addition, exercise habits before pregnancy and employment during pregnancy were positively associated meeting the PA guideline during pregnancy. The dietary intake differs among different age groups.

The median total PA of the participants was 56.3 METs-h/week, exceeding what was reported by a previous study conducted in Tianjin (approximately 50 METs-h/week) [19]. More than half of the participants met the international guideline for PA, more than what was reported in Tianjin (11%) [19] and Australia (44%) [20], but fewer than what was reported in the United States (66%) [21]. Most women in this study had exercise habits before pregnancy (approximately 60%), which could have contributed to the elevated total PA during pregnancy. As Bungum et al. [22] indicated, most women habituated to exercising would continue the habit in the pregnant period. Moreover, we found that household activity and occupational activity contributed the most to the physical activity energy expenditure, but sports/exercise contributed little, and the most reported exercise type was walking. These are consistent with previous studies [19, 23] which also reported slow walking as the most common form of exercise for pregnant women. Guelfi et al. [23] found that the levels of moderate and vigorous physical activity in Australian women was 6 times as much as in Chinese women when walking is not included. This may be related to the traditional view in China that pregnancy is a vulnerable period and requires rest and recuperation [24]. However, some studies showed that only moderate or vigorous PA appears beneficial with respect to maternal metabolic during pregnancy [11, 25, 26]. It suggests that higher intensity PA are required to improve the health for Chinese women.

The energy intake reported in this study is consistent with Liu et al.’s finding (mean = 2098 kcal/day) [27]. Although it may be adequate to meet the needs of pregnancy [5], most participants had an imbalanced diet. Fat comprised 41.7% of the mean energy intake (Chinese dietary reference: < 30%) [5], which is similar to previous studies revealing that Chinese women gain approximately 40% of their energy from fat [27, 28]. Overconsumption of oil may have contributed to high fat intake. This is supported by a previous study which shows that 60% of Chinese women consumed more oil than recommended by the Dietary Guideline for Chinese Residents [27]. In addition, women in China are traditionally encouraged to increase their consumption of meat or meat soup during pregnancy, which may have also contributed to high fat intake [29]. Some studies showed that maternal over-nutrition with a “high fat” diet predisposes the progeny to obesity and metabolic diseases [30, 31]. Yet, a recent review [31] has shown that the types of fatty acids consumed during pregnancy play an important role in normal fetal and postnatal development, and the effect of fat on the pregnancy outcome is different between the Western and Asian societies [32]. The effect of the type of fat on pregnancy outcomes among Chinese women needs to be further examined.

This study did not find a significant relationship between PA and dietary intake of pregnant women. Some studies have found a positive relationship or a synergistic effect between these two modifiable lifestyle factors in general adult populations [13, 14]. The inconsistency may be because when non-pregnant women have a high level of PA, they usually have high intrinsic motivation to improve health, which also leads to healthy dietary intake. However, in the traditional Chinese culture, women in pregnancy may be under pressure from family and friends and cultural taboos, which may contradict the recommendations from the authoritative guidelines of PA and dietary intake [19, 24].

Finally, the influencing factors of PA and dietary intake in Chinese women were identified. Employment during pregnancy and exercise habits before pregnancy were positively associated with the PA behavior for Chinese pregnant women. A previous study showed that Chinese women viewed that it is important to stop working during pregnancy [23]. In this paper, we also found that more than half of the women were unemployed during pregnancy and the percentage of unemployment was higher in later trimesters. Benefits such as improved mood and fitness can be gained from employment during pregnancy. Therefore, education of the benefits of employment during pregnancy is necessary. As described above, exercise habits before pregnancy was associated with the exercise behavior during pregnancy [22]. However, we also found that exercise habits before pregnancy and age were positively associated with fat intake (the similar result was shown in total calories). Since a study showed a positive correlation of PA with dietary fat intake [33], we analyzed this relationship further. We found the women older than 30 years were more likely to have exercise habits before pregnancy than women younger than 25 years, and there is no difference of gestational weight gain between them. It was interesting to find that women in different age groups may pay attention to different ways (increasing PA or reducing dietary intake) of weight control. Future health behavior interventions for Chinese pregnant women may need to consider the difference between age groups.

Our study has a few limitations. First, the data collection from only one city may make our findings less generalizable, although we chose a large city with both rural and urban regions. Second, the PA and dietary intake were self-reported by the pregnant women and subjective bias could be a concern. While this may possibly affect the absolute value of PA or dietary intake, it is not likely to affect the relative values of PA and dietary intake among groups and their relationships with the influence factors, assuming that all the participants were equally biased in self-reporting. This assumption should be checked in future research, though. Finally, since the study was cross-sectional, we could not test causal associations between the variables. Controlled experiments that examine the causal effect of PA and fat intake on pregnancy outcome are needed.

## Conclusions

This study revealed the characteristics of PA and dietary intake during pregnancy among Chinese women, showing that Chinese women performed relatively high total PA, but time spent on exercise was little, and their dietary intake was imbalanced, containing excessive calories from fat. Different from the general women population, the pregnant women’s PA behavior was not directly associated with their dietary intake. In addition, starting exercise before pregnancy may help women achieve adequate PA during pregnancy. Our findings also suggest that interventions for Chinese pregnant women may need to consider age difference. The revelation of the characteristics of PA and dietary intake during pregnancy and the identification of their associations during pregnancy for Chinese women may help to refine and improve intervention efforts on maternal and fetal health.

## Data Availability

The data sets used and/or analyzed during the current study are available upon reasonable request.

## Abbreviations

BMI: Body mass index
FFQ: The food frequency questionnaire
GWG: Gestational weight gain
PA: Physical activity
PPAQ: The pregnancy physical activity questionnaire

## References

[1] Mitanchez D, Chavatte-Palmer P (2018). Review shows that maternal obesity induces serious adverse neonatal effects and is associated with childhood obesity in their offspring. Acta Paediatr.

[2] Carreno CA, Clifton RG, Hauth JC, Myatt L, Roberts JM, Spong CY, Varner MW, Thorp JM, Mercer BM, Peaceman AM (2012). Excessive early gestational weight gain and risk of gestational diabetes mellitus in nulliparous women. Obstet Gynecol.

[3] Knudsen VK, Heitmann BL, Halldorsson TI, Sørensen TIA, Olsen SF (2013). Maternal dietary glycaemic load during pregnancy and gestational weight gain, birth weight and postpartum weight retention: a study within the Danish National Birth Cohort. Brit J Nutr.

[4] American College Of Obstetricians Gynecologists (2015). Physical activity and exercise during pregnancy and the postpartum period. Committee Opinion.

[5] Chinese Nutrition Society: Chinese Dietary Reference Intakes Handbook (2013). 2014.

[6] Insaf TZ, Strogatz DS, Yucel RM, Chasan-Taber L, Shaw BA (2014). Associations between race, lifecourse socioeconomic position and prevalence of diabetes among US women and men: results from a population-based panel study. J Epidemiol Commun H.

[7] McCullough LE, Mendez MA, Miller EE, Murtha AP, Murphy SK, Hoyo C (2015). Associations between prenatal physical activity, birth weight, and DNA methylation at genomically imprinted domains in a multiethnic newborn cohort. Epigenetics-Us.

[8] Jiang H, Qian X, Li M, Lynn H, Fan Y, Jiang H, He F, He G (2012). Can physical activity reduce excessive gestational weight gain? Findings from a Chinese urban pregnant women cohort study. Int J Behav Nutr Phys Act.

[9] Pei L, Kang Y, Zhao Y, Cheng Y, Yan H (2016). Changes in socioeconomic inequality of low birth weight and macrosomia in Shaanxi Province of Northwest China, 2010–2013. Medicine.

[10] Stuebe AM, Oken E, Gillman MW (2009). Associations of diet and physical activity during pregnancy with risk for excessive gestational weight gain. Am J Obstet Gynecol.

[11] Ehrlich SF, Sternfeld B, Krefman AE, Hedderson MM, Brown SD, Mevi A, Chasan-Taber L, Quesenberry CP, Ferrara A (2016). Moderate and vigorous intensity exercise during pregnancy and gestational weight gain in women with gestational diabetes. Matern Child Health J.

[12] Haakstad LAH, Voldner N, Henriksen T, Bø K (2007). Physical activity level and weight gain in a cohort of pregnant Norwegian women. Acta Obstet Gyn Scan.

[13] Gillman MW, Pinto BM, Tennstedt S, Glanz K, Marcus B, Friedman RH (2001). Relationships of physical activity with dietary behaviors among adults. Prev Med.

[14] Sauder KA, Starling AP, Shapiro AL, Kaar JL, Ringham BM, Glueck DH, Leiferman JA, Siega-Riz AM, Dabelea D (2016). Diet, physical activity and mental health status are associated with dysglycaemia in pregnancy: the healthy start study. Diabetic Med.

[15] Chasan-Taber L, Schmidt MD, Roberts DE, Hosmer D, Markenson G (2004). PS. F: development and validation of a pregnancy physical activity questionnaire. Med Sci Sports Exerc.

[16] Xiang M, Konishi M, Hu H, Takahashi M, Fan W, Nishimaki M, Ando K, Kim H, Tabata H, Arao T, Sakamoto S (2016). Reliability and validity of a Chinese-translated version of a pregnancy physical activity questionnaire. Matern Child Health J.

[17] Zhang H, Qiu X, Zhong C, Zhang K, Xiao M, Yi N, Xiong G, Wang J, Yao J, Hao L (2015). Reproducibility and relative validity of a semi-quantitative food frequency questionnaire for Chinese pregnant women. Nutr J.

[18] National Institute for Nutrition and Food Safety of Chinese Center for Disease Control and Prevention (2009). Chinese food composition table 2009.

[19] Zhang Y, Dong S, Zuo J, Hu X, Zhang H (2014). Y Z: physical activity level of urban pregnant women in Tianjin, China: a cross-sectional study. PLoS One.

[20] de Jersey SJ, Nicholson JM, Callaway LK, Daniels LA (2013). An observational study of nutrition and physical activity behaviours, knowledge, and advice in pregnancy. BMC Pregnancy Childbirth.

[21] Ning Y, Williams MA, Dempsey JC, Sorensen TK, Frederick IO, Luthy DA (2003). Correlates of recreational physical activity in early pregnancy. J Matern Fetal Neonatal Med.

[22] Bungum TJ, Peaslee DL, Jackson AW (2000). Exercise during pregnancy and type of delivery in nulliparae. JOGNN.

[23] Guelfi KJ, Wang C, Dimmock JA, Jackson B, Newnham JP, Yang H (2015). A comparison of beliefs about exercise during pregnancy between Chinese and Australian pregnant women. Bmc Pregnancy Childb.

[24] Lee DTS, Ngai ISL, Ng MMT, Lok IH, Yip ASK, Chung TKH (2009). Antenatal taboos among Chinese women in Hong Kong. Midwifery.

[25] Medek H, Halldorsson T, Gunnarsdottir I, Geirsson RT (2016). Physical activity of relatively high intensity in mid-pregnancy predicts lower glucose tolerance levels. Acta Obstet Gynecol Scand.

[26] Hopkins SA, Baldi JC, Cutfield WS, McCowan L, Hofman PL (2011). Effects of exercise training on maternal hormonal changes in pregnancy. Clin Endocrinol.

[27] Liu FL, Zhang YM, Parés GV, Reidy KC, Zhao WZ, Zhao A, Chen C, Ning CY, Zheng YD, PY W (2015). Nutrient intakes of pregnant women and their associated factors in eight cities of China: a cross - sectional study. Chin Med J.

[28] Gao H, Stiller C, Scherbaum V, Biesalski H, Wang Q, Hormann E, Bellows A (2013). Dietary intake and food habits of pregnant women residing in urban and rural areas of Deyang City, Sichuan Province, China. Nutrients.

[29] Raven JH, Chen Q, Tolhurst RJ, Garner P (2007). Traditional beliefs and practices in the postpartum period in Fujian Province, China: a qualitative study. BMC Pregnancy Childhealth.

[30] Szostak-Wegierek D (2014). Intrauterine nutrition: long-term consequences for vascular health. Int J Women's Health.

[31] Zhou D, Pan Y (2015). Pathophysiological basis for compromised health beyond generations: role of maternal high-fat diet and low-grade chronic inflammation. J Nutr Biochem.

[32] Mennitti LV, Oliveira JL, Morais CA, Estadella D, Oyama LM, Oller Do Nascimento CM, Pisani LP (2015). Type of fatty acids in maternal diets during pregnancy and/or lactation and metabolic consequences of the offspring. J Nutr Biochem.

[33] Boyle Raymond G., O'Connor Patrick J., Pronk Nicolaas P., Tan Agnes (1998). Stages of Change for Physical Activity, Diet, and Smoking among HMO Members with Chronic Conditions. American Journal of Health Promotion.

